# Male urogenital function after robot-assisted and laparoscopic total mesorectal excision for rectal cancer: a prospective cohort study

**DOI:** 10.1186/s12893-022-01592-1

**Published:** 2022-05-14

**Authors:** Bo Tang, Gengmei Gao, Shanping Ye, Dongning Liu, Qunguang Jiang, Junhua Ai, Xiong Lei, Jun Shi, Taiyuan Li

**Affiliations:** 1 Department of General Surgery, The First Affiliated Hospital of Nanchang University, Jiangxi 330006 Nanchang, China; 2 Nanchang University Medical College, Jiangxi Nanchang, China

**Keywords:** Da Vinci robot, Laparoscopic, Rectal cancer, TME, PANP, Urogenital function

## Abstract

**Background:**

Urogenital dysfunction is recognized as a serious complication affecting patient quality of life after rectal cancer surgery to treat rectal cancer; however, the studies focus on the urogenital function after robot-assisted rectal cancer surgery compared to laparoscopic surgery are limited.

**Methods:**

Male patients undergoing robotic total mesorectal excision (R-TME) or laparoscopic total mesorectal excision (L-TME) were prospectively enrolled. The International Prostate Symptom Score (IPSS) and the five-item version of the International Index of Erectile Function (IIEF-5) scale were used to compare the urogenital function of the two groups preoperatively and 3, 6, and 12 months postoperatively.

**Results:**

Eighty-nine patients who planned to undergo R-TME and L-TME were prospectively enrolled; 77 patients of these patients (86.5%) completed all questionnaires at all time points and were thus included in the final analysis. Of the included patients, 38 underwent R-TME and 39 underwent L-TME. There was no significant difference in age, BMI, American Society of Anesthesiologists (ASA) score, tumor location, neoadjuvant therapy, operation method, postoperative pathological results and adjuvant therapy between the two groups. Preoperative urogenital function was similar in both groups; however, the IPSS was significantly lower in R-TME patients than that in T-TME patients at 6 months and 12 months [(7.82 ± 2.25 vs. 9.95 ± 3.01, P = 0.006; 7.62 ± 2.5 vs. 9.12 ± 2.64, P = 0.012)]. IIEF-5 scores decreased 3 months after R-TME and L-TME surgery (14.87 ± 3.27 vs. 13.92 ± 3.62, p = 0.231) and then gradually increased; at 12 months, IIEF-5 scores were comparable to those at baseline in both groups. IIEF-5 scores were higher in R-TME patients than those in L-TME patients at 6 months (18.55 ± 3.45 vs. 16.75 ± 3.26, P = 0.021), but there was no significant difference between the two groups at 12 months (21.22 ± 3.06 vs. 19.95 ± 3.03, P = 0.071).

**Conclusions:**

The robotic approach for TME was associated with more rapid restoration of male urogenital function than the laparoscopic approach.

## Background

Rectal cancer is one of the most common malignant cancers worldwide, and its incidence is increasing in young people [1, 2]. Surgery is the main treatment for rectal cancer [3]. Total mesorectal excision (TME), proposed by Heald in 1982, greatly improved the survival rate and reduced recurrence in rectal cancer patients; however, after TME surgery, patients still exhibited higher urogenital dysfunction, which seriously affects the postoperative quality of life of patients [4, 5]. Therefore, Japanese researchers proposed TME with pelvic autonomic nerve preservation (PANP), which can preserve urogenital function to the maximum extent [6, 7].

At present, laparoscopy is widely used in the surgical treatment of rectal cancer, and its safety and oncologic outcomes are acknowledged [8, 9]. Laparoscopic rectal cancer surgery can reduce intraoperative blood loss, relieve postoperative pain and accelerate recovery from postoperative pain [10]; however, urogenital dysfunction after laparoscopic TME with PANP still persisted [11, 12].

In recent years, robotic surgery has gained greater popularity worldwide. This technique has several advantages over laparoscopic surgery, including an immersive three-dimensional view of the surgical field, better surgical dexterity, and a stable camera platform. Such innovative technology can alleviate some of the maneuverability and visibility challenges that surgeons encounter in narrow pelvic cavities [13]. Patients who undergo robotic surgery have better short-term outcomes and similar long-term outcomes to those who underwent laparoscopic surgery [13–15]; however, the studies focus on the urogenital function after robot-assisted rectal cancer surgery compared to laparoscopic surgery are limited [16–18].

Therefore, in this study, we evaluated urogenital function at several time points in male patients who underwent robotic-assisted or laparoscopic surgery for rectal cancer to determine which surgery had better urogenital function outcomes.

## Patients and methods

### Participants

This was a single-center, prospective, cohort study from the First Affiliated Hospital of Nanchang University in Jiangxi, China. Male patients with rectal cancer, tumors located within 12 cm of the anal verge, age ≤ 60 years, and normal urogenital function before surgery and who underwent robotic or laparoscopic surgery were included. The exclusion criteria included emergency operations, patients with distant metastases, a history of previous pelvic organ operations, or conversion to laparotomy, and patients that refused to join the study. The choice of surgical approach (robotic or laparoscopic) was determined in accordance with the wishes of the patient. The study received ethical approval from the First Affiliated Hospital of Nanchang University, and all patients provided informed consent for participation in the study.

### Surgical procedures

All operations were performed by one surgeon. All patients enrolled in the study underwent TME with PANP utilizing a medial-to-lateral approach. Lymph node dissection was performed to the root of the inferior mesenteric artery (IMA). High or low ligation was performed according to the length of the colon and rectum. All rectal cancer resections adhered to the principles of TME. For PANP, the superior hypogastric plexus (SHP) was preserved at the root of the IMA. The hypogastric plexus (HP) and pelvic splanchnic nerves (PSN) were preserved when dissecting the mesorectum posteriorly, the inferior hypogastric plexus (IHP), PSN and the neurovascular bundle (NVB) of its branches were preserved when dissecting the mesorectum laterally and anteriorly respectively. Linear stapler devices were used to transect the rectum 1–2 cm below the tumor. The specimen was extracted through a 4- to 5-cm mini-laparotomy in the lower abdomen with a wound protector. The bowel was anastomosed using a circular stapler. Ileostomy was conducted according to the risk factors for anastomotic leakage and reversed at 3 months after surgery. Abdominoperineal resection was performed if the distal resection margin of 1–2 cm cannot be confirmed with negative in low anterior resection.

### Assessment of urogenital function

The International Prostate Symptom Score (IPSS) [19] was used to assess urinary function. The IPSS includes seven items, each of which is assigned a score from 1 to 5 (for a maximum score of 35): emptying, frequency, intermittency, urgency, weak stream, hesitancy, nocturia, Higher scores indicate more severe urinary dysfunction.

The five-item version of the International Index of Erectile Function (IIEF-5) scale [20] was used to assess male erectile function. It consists of five questions: confidence in erectile function, success rate of insertion after erection, maintaining an erection, success rate of sexual intercourse and satisfaction after sexual intercourse. Each item is assigned a score from 0 to 5 points, with a total score of 25 points; higher scores indicate better sexual function.

Both questionnaires were administered preoperatively and 3, 6, and 12 months postoperatively.

### Statistical analysis

Statistical analyses were performed using SPSS 24.0. Categorical variables were compared with Chi-square tests; continuous variables were compared with Student’s t tests or Mann–Whitney U tests. P values < 0.05 were considered statistically significant.

## Results

From June 2018 to July 2020, 89 patients who were going to undergo R-TME or L-TME at the First Affiliated Hospital of Nanchang University were prospectively enrolled in this study. Seventy-seven patients (86.5%) who completed all the questionnaires at all time points were included in the final analysis: 38 underwent surgery with a robotic approach and 39 underwent laparoscopy. The two groups did not significantly differ in age, body mass index (BMI), American Society of Anesthesiologists (ASA) score, tumor location, neoadjuvant therapy, operation method, postoperative pathological results and adjuvant therapy (Table 1).

**Table 1:** Patient and procedure characteristics of two groups

Variables	R-TME (n = 38)	L-TME (n = 39)	P value
Age (years)	47.75 ± 9.62	46.01 ± 9.37	0.424
BMI (kg/m^2^)	21.34 ± 2.67	22.03 ± 2.51	0.246
ASA score n(%)			0.301
I	22(57.9%)	27(69.2%)	
II	16(42.1%)	12(30.8%)	
Tumor location (cm)	6.33 ± 2.21	5.82 ± 2.43	0.339
Neoadjuvant therapy n (%)	9(23.7%)	11(28.2%)	0.651
Operation method n (%)			0.591
LAR	32(84.2%)	31(79.5%)	
APR	6(15.8%)	8(20.5%)	
Stoma n (%)	8(21.1%)	7(17.9%)	0.731
Tumor differentiation n (%)			0.826
Well	5(13.2%)	6(15.4%)	
Moderate	25(65.8%)	23(59.0%)	
Poor	8(21.0%)	10(25.6%)	
Tumor stage n (%)			0.367
T1	3(5.3%)	3(7.7%)	
T2	10(26.3%)	7(17.9%)	
T3	22(57.9%)	24(61.5%)	
T4	3(7.9%)	5(12.8%)	
Nodal stage n (%)			0.761
N0	28(73.7%)	30(76.9%)	
N1	7(18.4%)	6(15.4%)	
N2	3(7.9%)	3(7.7%)	
Adjuvant therapy n (%)	13(34.2%)	15(38.5%)	0.114

*BMI* body mass index, *ASA* American Society of Anesthesiologists, *LAR* low anterior resection, *APR* abdominoperineal resection

### Urinary function

**Fig. 1:**
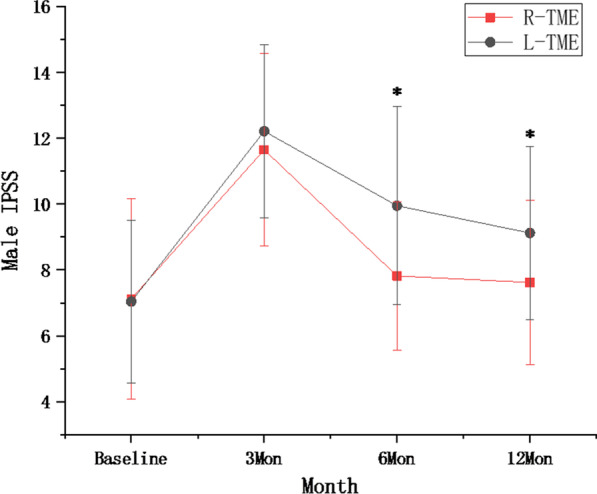
IPSS between the two groups, *indicated significant difference between two groups at the time point

The preoperative total IPSS was similar in both groups, but the IPSS was significantly lower in the robotic group than in the laparoscopic group at 6 months and 12 months [(7.82 ± 2.25 vs. 9.95 ± 3.01, P = 0.006; 7.62 ± 2.5 vs. 9.12 ± 2.64, P = 0.012)], (Table 2; Fig. 1).

**Table 2:** IPSS between the two groups

	R-TME (n = 38)	L-TME (n = 39)	P value
Baseline	7.12 ± 3.05	7.04 ± 2.48	0.880
3Mon	11.65 ± 2.93	12.21 ± 2.62	0.379
6Mon	7.82 ± 2.25	9.95 ± 3.01	0.006
12Mon	7.62 ± 2.5	9.12 ± 2.64	0.012

*IPSS* International Prostate Symptom Score, *TME* total mesorectal excision

### Sexual function

**Fig. 2:**
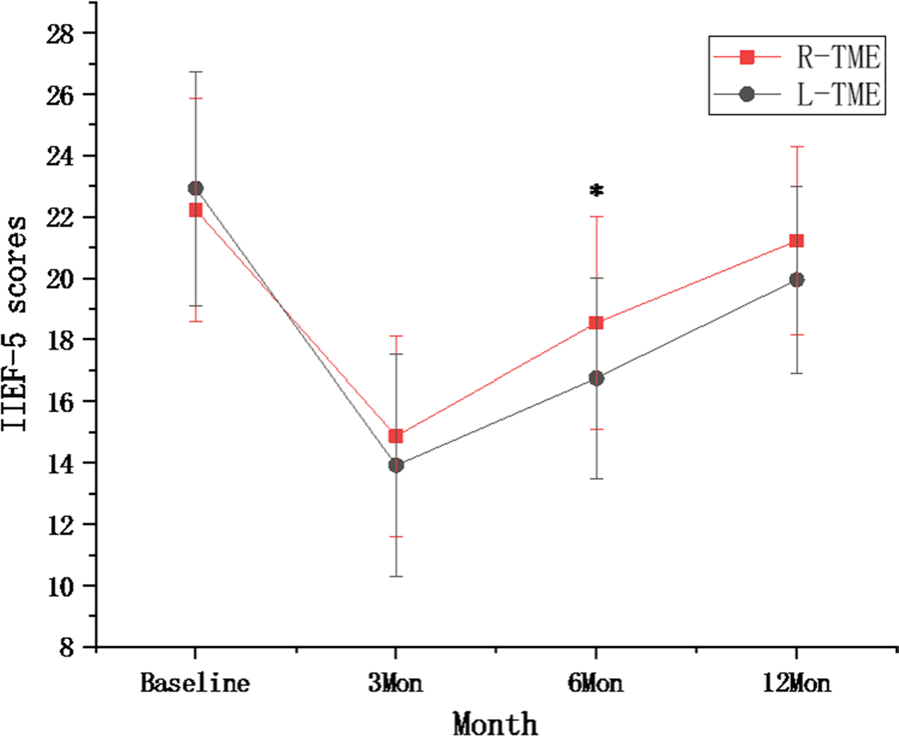
IIEF-5 scale between the two groups, *indicated significant difference between two groups at the time point

The baseline IIEF-5 scores of the two groups were similar. IIEF-5 scores decreased 3 months after surgery and then gradually increased; by 12 months, scores were comparable to those at baseline scores in both groups. However, IIEF-5 scores were higher in the robotic group than in the laparoscopic group at 6 months (18.55 ± 3.45 vs. 16.75 ± 3.26, P = 0.021), but there was no significant difference between the two groups at 12 months (21.22 ± 3.06 vs. 19.95 ± 3.03, P = 0.071), (Table 3; Fig. 2).

**Table 3:** IIEF-5 scores between the two groups

	R-TME (n = 38)	L-TME (n = 39)	P value
Baseline	22.23 ± 3.65	22.93 ± 3.82	0.414
3Mon	14.87 ± 3.27	13.92 ± 3.62	0.231
6Mon	18.55 ± 3.45	16.75 ± 3.26	0.021
12Mon	21.22 ± 3.06	19.95 ± 3.03	0.071

*IIEF-5* International Index of Erectile Function

## Discussion

Given the continuous advances in early diagnosis, surgical techniques chemo-radiotherapy, targeted therapy and immunotherapy, the survival of patients with rectal cancer has greatly improved; however, urogenital dysfunction resulting from rectal cancer surgery is a major problem affecting their quality of life [5]. PANP provides a theoretical basis for improving postoperative urogenital dysfunction. In this study, we found that using the robotic approach was associated with more rapid restoration of male urogenital function than the laparoscopic approach.

Age, tumor location, use of preoperative radiotherapy, the operation method and the stoma can affect postoperative urogenital function [5, 21–23]. Havenga K reported that [24] more than 86% of patients under 60 years of age were sexually active, while only 60% of patients over 60 years of age were sexually active; thus, in this study, we only included patients who were sexually active one month before surgery and were younger than 60 years old. Preoperative pelvic radiotherapy can cause inflammatory pelvic reactions, leading to injury of the pelvic nerve and fibrosis of the genitals. Tumor location determines the type of operation. The lower the dissecting level of the TME is, the greater the probability of damaging the pelvic plexus and NVB. Abdominoperineal excision and stoma have multiple physiological and psychological effects on patients, leading to postoperative urogenital dysfunction. In our study, patients who underwent neoadjuvant therapy, tumor location, type of operation, and the stoma were no significant difference between the two groups. Therefore, we further evaluated the influence of the robot and laparoscopic operation platforms on postoperative urogenital function.

Our results showed that male urinary function decreased at 3 months in the two groups and then gradually improved. Urinary function recovered to baseline scores after 6 months in the robotic group, while in the laparoscopic group, urinary function still had not recovered after 12 months. This finding was similar to the studies of Kim et al. [17, 25], robotic surgery can provide more rapid recovery of urinary function. However, Park and ROLARR [26, 27] found that robotic surgery and laparoscopic surgery had similar effects on postoperative urinary function. When TME is performed in rectal cancer surgery, nerve damage usually occurs during ligation of the IMA, and during posterior, lateral and anterior rectal dissection [28]. Intraoperative traction, the heat of the platform and postoperative inflammation can cause temporary nerve damage, which can be compensated for and recovers more quickly; in contrast, intraoperative electrocoagulation and ligation lead to permanent nerve damage. The enlarged visual field, dexterity of surgical instruments and use of electric scissors in robotic surgery can not only accurately perform TME but also prevent permanent nerve injury caused by unclear or blind separation.

In this study, IIEF-5 scores decreased 3 months after surgery and then gradually increased; at 12 months, scores were comparable to those at baseline in both groups. However, IIEF-5 scores were higher in the robotic group than in the laparoscopic group at 6 months, which was in line with previous studies [17, 25, 27, 29]. Erectile function is restored to preoperative levels more quickly after robotic surgery. Erectile function is mainly dependent on the pudendal nerve, pelvic plexus and the NVB of its branches; it is easy to damage the pelvic plexus and NVB during laparoscopic surgery due to insufficient or excessive traction and difficulty in identifying the nerve and the operation plane in the narrow pelvis. The enlarged three-dimensional view provided by the robot can clearly identify the Denonvilliers’ fascia during anterior rectal dissections, and its unique operating platform can maintain stable tension when performing lateral and posterior rectal dissection, which effectively protects the pelvic plexus and NVB at 2 o’clock and 10 o’clock in the plane of the seminal vesicle.

Our study also had some limitations. First, this study was not a randomized controlled trial; thus, the validity of our data may be weaker. Second, the evaluation indices of genitourinary function in this study were subjective and easily affected by psychological factors, making it difficult to distinguish whether symptoms were caused by psychological factors or physiological factors. Third, due to ethical and cultural factors, the sample size of this study was small and did not evaluate female genitourinary function.

## Conclusions

Our study found that the robotic approach for TME was associated with a more rapid restoration of male urogenital function compared to that of the laparoscopic approach. Multicenter randomized controlled trials are needed to confirm the advantages of robotic surgery for rectal cancer.

## Data Availability

The data that support the findings of this study are available from the authors, but restrictions apply to the availability of these data, which were used under license for the current study, and so are not publicly available. Data are however available from the authors upon reasonable request and with permission of ethics committee First Affiliated Hospital of Nanchang University.

## Abbreviations

TME: Total mesorectal excision
PANP: Pelvic autonomic nerve preservation
SHP: Superior hypogastric plexus
IMA: Inferior mesenteric artery
HP: Hypogastric plexus
PSN: Pelvic splanchnic nerves
NVB: Neurovascular bundle
IPSS: International Prostate Symptom Score
IIEF: International Index of Erectile Function
BMI: Body mass index
ASA: American Society of Anesthesiologists
